# In-office dental bleaching in adolescents using 6% hydrogen peroxide with and without gingival barrier: a randomized double-blind clinical trial

**DOI:** 10.1590/1678-7757-2023-0416

**Published:** 2024-03-14

**Authors:** Taynara de Souza CARNEIRO, Michael Willian FAVORETO, João Pedro Ferreira RODRIGUES, Elisama SUTIL, Gabrielle Gomes CENTENARO, Isabela de Matos de FREITAS, Alessandra REIS, Laura Ceballos GARCÍA, Alessandro Dourado LOGUERCIO

**Affiliations:** 1 Universidade Estadual de Ponta Grossa Departamento de Dentística Restauradora Ponta Grossa Brasil Universidade Estadual de Ponta Grossa, Departamento de Dentística Restauradora, Ponta Grossa, Brasil.; 2 Universidad Rey Juan Carlos Facultad de Ciencias de la Salud IDIBO Madrid España Universidad Rey Juan Carlos, Facultad de Ciencias de la Salud, IDIBO, Madrid, España.

**Keywords:** Tooth bleaching agents, Hydrogen peroxide, Gingiva, Clinical trial, Adolescent, RCT, Teeth whitening

## Abstract

At low concentrations used for in-office bleaching gels, such as 6% HP, gingival barrier continues to be performed. If we take into account that, in the at-home bleaching technique, no barrier is indicated, it seems that the use of a gingival barrier fails to make much sense when bleaching gel in low concentration is used for in-office bleaching.

**Objective:**

This double-blind, split-mouth, randomized clinical trial evaluated the gingival irritation (GI) of in-office bleaching using 6% hydrogen peroxide (HP) with and without a gingival barrier in adolescents, as well as color change and the impact of oral condition on quality of life.

**Methodology:**

Overall, 60 participants were randomized into which side would or would not receive the gingival barrier. In-office bleaching was performed for 50 minutes with 6% HP in three sessions. The absolute risk and intensity of GI were assessed with a visual analogue scale. Color change was assessed using a digital spectrophotometer and color guides. The impact of oral condition on quality of life was assessed using the Brazilian version of the Oral Health Impact Profile (α=0.05).

**Results:**

The proportion of patients who presented GI for the “with barrier” group was 31.6% and for the “without barrier” group, 30% (p=1.0). There is an equivalence for the evaluated groups regarding GI intensity (p<0.01). Color change was detected with no statistical differences (p>0.29). There was a significant impact of oral condition on quality of life after bleaching (p<0.001).

**Conclusions:**

The use or not of the gingival barrier for in-office bleaching with 6% HP was equivalent for GI, as well as for bleaching efficacy, with improvement in the impact of oral condition on quality of life.

## Introduction

Adolescents are concerned about social acceptance and their emotional well-being and self-esteem can be improved by dental aesthetic procedures.^1^ Among them, dental bleaching is considered a less invasive technique with positive effects on these patients’ quality of life and aesthetic self-perception.^2^

Dental bleaching is very effective both in the case of intrinsic and extrinsic discolorations^3,4,5^ and can be done in various ways. In-office bleaching techniques that use high or medium concentrations of hydrogen peroxide (HP) (20% to 40%) provide faster results.^6,7^ However, several adverse effects have been observed, among which are tooth sensitivity and gingival irritation (GI).^8^

**Figure 1:**
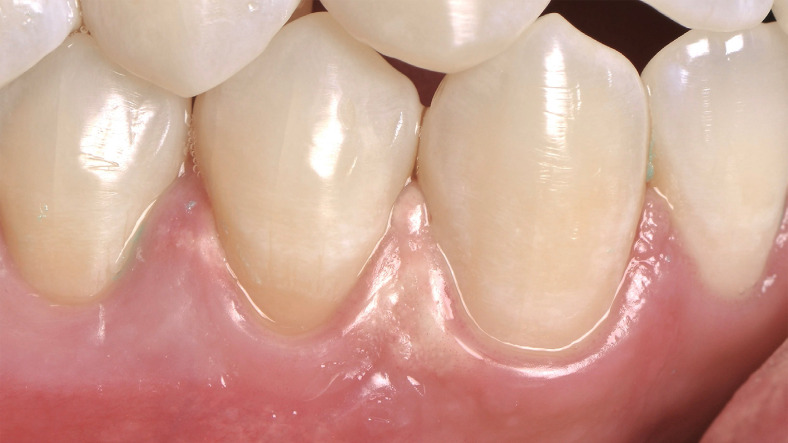
Appearance of gingival irritation with burns in a patient subjected to 35% hydrogen peroxide bleaching gel during in-office bleaching. This concentration of hydrogen peroxide was not evaluated in the present study

GI is less prevalent and is directly related to the operator’s process. The operator should have complete mastery of the technique. Failure to correctly light-cure the gingival barrier or carelessness in application^8^ may cause this adverse effect. GI can manifest itself as mild discomfort leading to burns and ulcerations^9^ due to the direct contact of soft tissues with the bleaching gel, being exacerbated according to the time and concentration (Figure 1) of the bleaching gel in contact with the gingival area.^10^ Because of this, when in-office bleaching with high concentrations of HP is performed, it is essential to use light-curing barriers to protect the soft tissue.^6^

Low/medium^13^ concentrations of bleaching gel (6-20%) have been developed by manufacturers with the intention to reduce the adverse effects of in-office dental bleaching. Recently, several clinical studies have shown excellent bleaching efficacy and fewer adverse effects when low concentrations of HP were used.^2,12,13^ These aspects appear particularly crucial in younger patients, such as adolescents. The most recommended technique for them thus far has been at-home bleaching due to its low concentration.^14^ This is especially relevant because adolescents typically have more permeable teeth given their maturation level.^15^ However, studies have indicated that adolescents encounter challenges in removing excess bleaching gel from trays and find them somewhat difficult to use,^16^ making low concentration in-office bleaching a good option to avoid this problem. In light of these findings, low-concentration in-office bleaching emerges as a viable option to address these issues.

However, despite the low concentrations used for in-office bleaching gels, such as 6% HP, gingival barriers continue to be required.^12,13,17^ No barrier is indicated for at-home bleaching techniques (up to 10% HP) regardless of the delivery method^18^ because few patients have reported GI.^19,20^ Therefore, it seems that the use of a gingival barrier fails to make much sense for the use of bleaching gel in low concentrations for in-office bleaching. However, to the extent of the authors’ knowledge, no clinical studies have evaluated this hypothesis. Note that removing the application and light-curing step from the gingival barrier may make the clinical procedure faster, more practical, and, therefore, more economically viable.

Therefore, the aim of this double-blind, controlled, split-mouth, randomized equivalence clinical trial was to evaluate GI during in-office bleaching with 6% HP in adolescents with or without the use of the gingival barrier, as well as the efficacy of bleaching and the effect of the oral condition on patients’ quality of life when subjected to low concentrations of in-office bleaching. The following null hypotheses were tested: 1) the use (or lack of use) of a gingival barrier will fail to affect the absolute risk and intensity of GI induced by in-office bleaching; 2) the use (or lack of use) of a gingival barrier for in-office bleaching will fail to affect color change; and 3) a low concentration of bleach will fail to affect the influence of oral condition on quality of life.

## Methodology

### Study design

This was a randomized, double-blind (evaluators and participants), split-mouth, and equivalence study. After approval of this clinical trial by the ethics committee at the State University of Ponta Grossa, PR, Brazil (4.935.724), it was registered in the Brazilian Clinical Trials Registry (RBR-8q6mfhc). This study followed the Consolidated Standards of Reporting Trials protocol with the extension for noninferiority and equivalence trials and within-person designs,^21^ and it was performed in the clinics of the school of dentistry at the State University of Ponta Grossa, PR, Brazil from September 2021 to February 2022.

### Recruitment and eligibility criteria

Participants were recruited on social media. This approach was carried out by sharing posts on both the Instagram feed and stories of the @bleachingbond research group user account. Additionally, the authors and other members of the research team reposted this content to further amplify its reach. Participants who met the eligibility criteria, before being included in the study, agreed to participate and their guardians read and signed informed consent forms.

Inclusion criteria were adolescents aged from 12 to 16 years with vital teeth free from caries lesions, periodontal disease, and endodontic treatment, with both mandibular canines presenting A2 or darker color according to the VITA Classical guide (VITA Zahnfabrik, Bad Säckingen, Germany), and in good general and oral health. Participants who were on chronic medication, who had undergone previous tooth bleaching, had previous tooth sensitivity, used fixed orthodontic appliances or prostheses, had parafunction, gingival recession, discoloration due to fluorosis or tetracycline, were pregnant or breastfeeding, had visible cracks in the teeth, or were smokers were excluded.^2,12^

### Sample size calculation

The primary outcome of this study was to assess the absolute risk of GI due to in-office bleaching with 6% HP. Based on a pilot study (*data not shown*), a total of 10% GI was reported. For a control group with 10% GI risk and an equivalence threshold of 20%, a minimum of 49 participants per group was required with 90% study power and 5% alpha. A sample size of 60 participants was used to compensate for any losses at follow-up.

### Random sequence generation and allocation concealment

Simple randomization was performed in an online software (*www.sealedenvelope.com*) by a person outside the research protocol. Randomization was placed in a sealed and opaque envelope, sequentially numbered, and only revealed five minutes before the start of the bleaching procedure. Treatment of the lower right hemi-arch was decided based on the information within the envelope (with or without gingival barrier) while the alternative treatment was applied to the other hemi-arch. This procedure was conducted by a researcher indirectly involved in any of the experimental phases.

### Blinding

This was a double-blind study in which the evaluator and the participant were unaware of group assignment. The color evaluator was kept from participating in the process of randomization and implementation of the study and participant received a simulated application and photopolymerization of the gingival barrier in the hemiarch without treatment. Due to differences between bleaching procedures, the operator cannot be blinding.

### Study intervention

Overall, three dentists with more than five years of clinical experience performed the bleaching procedure. Before bleaching, participants underwent prophylaxis to remove extrinsic stains. The ArcFlex retractor (FGM, Joinville, SC, Brazil) was placed, which promotes retraction of the lips, cheeks, bite rest, and tongue control. Then, the operator opened the randomization envelope to visualize the group in which the patient’s right hemi-arch was (with or without a gingival barrier). The procedure was performed with light-curing resin Top Dam (FGM, Joinville, SC, Brazil). Before performing the gingival barrier, the region in which the application would be performed was dried. The gingival barrier was applied to the corresponding teeth up to the second premolar region, being applied with a thickness of approximately 1 mm over the gingiva and gingival papillae, obtaining full coverage with a good protection field for the procedure. The barrier was light-cured for 20 seconds using an LED unit set at 1400 mW/cm^2^ (Valo high power mode, Ultradent, South Jordan, UT, USA), and light-curing was performed immediately after application of the gingival barrier to prevent any possible irritation. Then, the bleaching gel Whiteness HP Automixx 6% (FGM, Joinville, SC, Brazil) was applied with a tip. Each session lasted 50 minutes, with three sessions with an interval of seven days between them.^2^ Participants were instructed to use dentifrices without desensitizers and without bleaching agents for daily brushing.

### Gingival irritation (GI) evaluation

To assess the absolute risk and intensity of GI, participants were instructed to record their GI using the Visual Analogue Scale (VAS; 0-10),^19^ in which 0 = no irritation and 10 = severe irritation. Participants were instructed to record their GI even if there was no irritation, marking with a vertical line the value corresponding to the intensity of their GI, immediately after, up to 1 hour after, up to 24 hours after, and up to 48 hours after the bleaching session, with the right and left hemiarches always being evaluated separately, which was (the VAS with the marking) later measured in cm with the aid of a millimeter ruler. Regarding risk, any value greater than zero was considered as a presence of GI, described in percentages, and intensity was measured in cm (1^st^, 2^nd^, and 3^rd^session and worst overall scenario [worst measured value for the three weeks]). Participants received all the guidelines for a better perception and greater description of results regarding GI, which may present pain and discomfort.

### Color evaluation

Color was registered before and after 30 days of the end of treatment for all evaluated parameters, with a measurement being taken at each evaluation moment. The two evaluators were calibrated before the study, presenting superior color-matching competency according to the ISO/TR 28642.^22^ In case of disagreement during the evaluation, they needed to reach a consensus before the participant was dismissed. Color evaluation was done in a room under artificial lighting conditions without interference from outside light.

Color evaluation was also performed using the objective method (Vita Easyshade spectrophotometer; Vita Zahnfabrik). The Vita Easyshade spectrophotometer (Vita Zahnfabrik) was used, according to the CIEL*a*b* system^2,12,13,17^, where L* represents the lightness value from 0 (black) to 100 (white) and a* and b* represent the color, in which a* is the measurement along the green-red coordinate and b* is the measurement along the blue-yellow coordinate. These values were provided by a spectrophotometer that was calibrated before each measurement per patient. To standardize the measurement of objective color, an impression of the lower arch of participants, with condensation silicone (Perfil, Coltene, Altstätten, Switzerland), was performed to make a guide for the lower anterior teeth. The matrix was perforated with the aid of a 6-mm diameter circular scalpel (Biopsy Punch, Miltex, York, NJ, USA), similar to the active tip of the Vita Easyshade spectrophotometer, in the vestibular region and middle third of the lower right and left canines. In total, three measurements were performed for each tooth and average values were used for statistical purposes.

The difference between the colors registered before and 30 days after the end of the treatment was calculated using the formulas CIELab:ΔE_ab_ = [(ΔL*)^2^ + (Δa*)^2^ + (Δb*)^2^]^1/2^,^23^ CIEDE 2000 ΔE 00 = [(Δ_L_’/k_L_S_L_)^2^ + (ΔC’/k_C_S_C_)^2^ + (ΔH’/k^H^S^H^)^2^ + RT (ΔC’/K_C_S_C_) (ΔH’/ K_H_S_H_)]^1/2^,^24^ where ΔL’, ΔC’, and ΔH’ are the CIELab metric lightness, chroma, and hue differences, respectively, R_T_ is the rotation function that accounts for the interaction between chroma and hue differences. S_L_, S_C_, and S_H_ are the weighting functions for the lightness, chroma, and hue components, respectively. The values calculated for these functions vary according to the positions of the sample pair being considered in CIELab color space. The k_L_, k_C_, and k_H_ values are the parametric factors to be adjusted according to different viewing parameters,^24^ and Whiteness Index for Dentistry was calculated according to the following formula: WI_D_ = 0.551×L−2.324×a−1.1×b. Moreover, changes in WI_D_ caused by each step were calculated by subtracting the values observed at each assessment time from those calculated in the prior step (ΔWI_D_).^25^ The following color change acceptability limits were used: CIELab 2.7,^26^ for CIEDE 2000 1.8,^26^ and Whiteness Index for Dentistry 2.6.^27^

Also, color evaluation was performed by the subjective method (Vita Classical and Vita Bleachedguide 3D-MASTER; Vita Zahnfabrik). The value-oriented Vita Classical color scale (Vita Zahnfabrik) consists of 16 color guides arranged from highest (B1) to the lowest (C4)^2,12,17^ and the Vita Bleachedguide 3D-MASTER scale (Vita Zahnfabrik) is a proper tooth bleaching scale containing lighter colored tabs arranged from the highest (0M1) to the lowest value (5M3).^2^ The evaluation of color change was performed by the variation of Vita scale units (ΔSGU) subtracted from the initial color and the unit of color reached after bleaching, organized by value,^12,17^ in the right and left canines.

### Impact of oral condition on quality of life

The impact of oral condition on quality of life was evaluated by the Brazilian version of the abbreviated form of the Oral Health Impact Profile (OHIP-14), which contains 14 questions.^28^ Participants were instructed to respond by marking the questions (0-4) with an X, where zero = never, one = rarely, two = sometimes, three = repeatedly, and four = always. The scale was delivered to be answered before the start of bleaching and after the end of the whole treatment. Participants received the questionnaire and answered it without any intervention from the evaluators and without a time limit for completion.

### Statistical analysis

Analysis followed the intention-to-treat protocol and involved all participants (who were randomly assigned).^29^ The statistician was blind to the assessment of the groups. The absolute risk of gingival irritation of both groups was compared using the McNemar test. Odds ratios were also calculated, as were 95% confidence intervals and Spearman’s correlation. Overall, two one-sided t-tests for paired samples (TOST-P) were used to test the equivalence of the study groups at the different assessment points for gingival irritation. The Student’s paired t-test and Pearson’s correlation were calculated for intensity of gingival irritation to detect differences between groups for each evaluation (1^st^, 2^nd^, and 3^rd^sessions and worst overall scenario). Color change between groups was compared using the Student’s paired t-test for the different instruments. The impact of oral condition on quality of life was compared using using Student’s paired t-test before and after bleaching. In all statistical tests, alpha was preset to 5%.

## Results

### Characteristics of included participants

**Figure 2:**
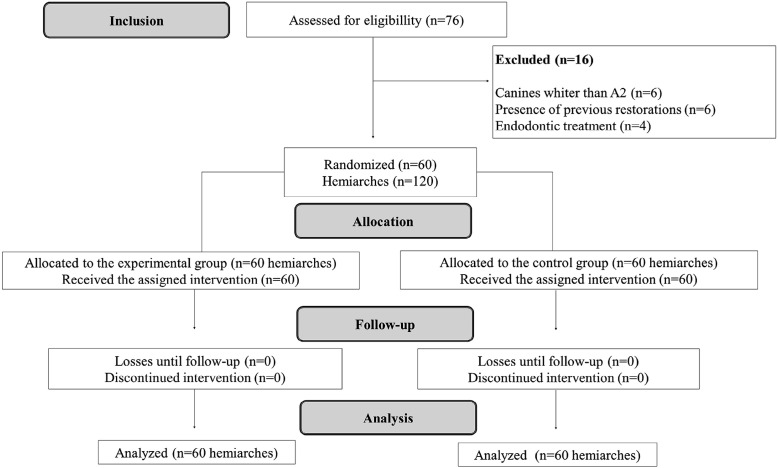
The CONSORT Flow Diagram of study design phases, including enrollment and allocation criteria

In total, 76 participants were examined, 60 of which were included in this clinical study (Figure 2). Table 1 describes the initial color of participants’ teeth and the distribution of their genders and ages with no loss of participants in the follow-up of this study.

**Table 1:** Baseline characteristics of the participants included in this clinical trial

Groups (number of patients)	With barrier (n = 60)	Without barrier (n = 60)
Baseline color (SGU; mean ± SD)*	10.0±2.7	10.6±2.1
Baseline color (WI_D_; mean ± SD)*	15.5±6.7	14.9±6.6
Gender (female; %)	40 (67%)	
Average age (years; female/male)	14.6	

*Abbreviations: SGU, shade guide unit measured by the Vita Classical scale; WI_D_, Whiteness Index for Dentistry measured by a Vita Easyshade spectrophotometer.

### Gingival irritation

**Figure 3:**
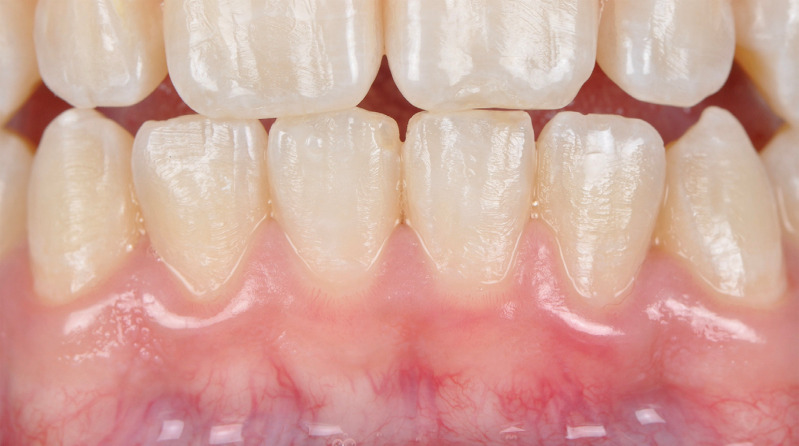
Appearance of gingival irritation in a patient subjected to 6% hydrogen peroxide bleaching gel during in-office bleaching. Despite the nearly imperceptible nature of gingival irritation, the patient reported experiencing irritation in the gingival area below teeth #31 and 41

GI (Figure 3) was reported by 30.8% of participants. While for the group with barrier, 31.6% of participants felt some discomfort, for the group without barrier, this discomfort was reported by 30% of participants. In relative terms, the odds ratio for irritation was 0.9 (0.4 to 2.0; Table 2), thus failing to reach statistical significance (*p*=1.0). Spearman’s correlation coefficient for pairs of binary data was moderate and significant (r=0.75; *p*<0.001).

**Table 2:** Matched tabulation of the absolute risk of gingival irritation for both groups along with the odds ratio and 95% confidence intervals (CI)

		Without barrier			Odds ratio
		Positive	Negative	Total	(95% CI)
**With barrier**	**Positive**	15	4	19	0.9 (0.4 to 2.0)
	**Negative**	3	38	41	
	**Total**	18	42	60	

*Mc Nemar test (p=1.0); Spearman's correlation between paired data (r=0.75; p-value <0.001).

Regarding intensity of GI, no significant difference between both groups was observed in the first (*p*=0.67), second (*p*=0.79), and third sessions (*p*=0.29), as well as in the overall worst-case scenario (*p*=0.77; Table 3). The mean difference in intensity of GI averaged below 0.3, which was far from clinically important. Irritation was positively correlated in both groups (Table 3). Pearson’s correlation was 0.83 (*p*<0.001) for the worst-case scenario in the first and second sessions, 0.75 (*p*<0.001) for the worst-case scenario for the third session, and 0.84 (*p*<0.001) for the overall worst-case scenario (Table 3).

**Table 3:** Means and standard deviations of the intensity of gingival irritation for both groups, mean differences (95% confidence interval [CI]), and correlation coefficients

Main factor time	With barrier	Without barrier	Mean difference (95% CI)	Equivalence [p-value]	p-value**	Correlation coefficient [p-value]
First Session	0.2±0.6	0.2±0.7	0.0 (-0.1 to 0.1)	Yes; p<0.01	0.67	0.83; p<0.0001
Second Session	0.1±0.3	0.1±0.3	0.0 (0.0 to 0.0)	Yes; p<0.01	0.79	0.83; p<0.0001
Third Session	0.0±0.2	0.0±0.1	0.0 (0.0 to 0.0)	Yes; p<0.01	0.29	0.75; p<0.0001
Worst overall scenario	0.3±0.7	0.3±0.7	0.0 (-0.1 to 0.1)	Yes; p<0.01	0.77	0.84; p<0.0001

* The p-value reported is the larger of the two p-values from the upper and lower one-sided tests (TOST test); **Paired t-test.

### Color change

Table 1 demonstrates that the initial tooth color averages in both groups were similar. After bleaching (Table 4), color change was detected for all evaluated parameters. Approximately, four units in the Vita Classical and Vita Bleachedguide scale, eight units in the ΔEab, five units in ΔE_00_, and 10 units in the ΔWID were observed, regardless of groups (Table 4). Comparison of both groups showed no significant difference in bleaching for none of the evaluated parameters (*p*>0.46; Table 4).

**Table 4:** Means and standard deviations in color change and mean differences (95% confidence intervals [CI]) baseline vs. one month

Color evaluation tool	Groups		Mean difference (95% CI)	p -value*
	With barrier	Without barrier		
CIELab (ΔE_ab_)	8.5±6.2	8.1±4.9	0.3 (-1.1 to 1.8)	0.64
CIEDE 2000 (ΔE_00_)	5.6±4.4	5.2±3.0	0.4 (-0.7 to 1.5)	0.46
Whiteness Index for Dentistry (ΔWI_D_)	10.4±6.1	10.1±5.9	0.3 (-1.3 to 2.0)	0.68
Vita Classical (ΔSGU)	3.8±2.3	3.7±2.3	0.0 (0.0 to 0.1)	0.56
Vita Bleachedguide (ΔSGU)	3.7±2.1	3.6±2.1	0.0 (-0.1 to 0.1)	0.48

*Paired t-test.

### Impact of oral condition on quality of life

When the impact of oral condition on quality of life was evaluated before and after the bleaching procedure, there was a significant impact of oral condition on quality of life after bleaching (*p*<0.001; Table 5).

**Table 5:** Medians and standard deviations for the Oral Health Impact Profile

	Means ± SD		Mean Difference (95% CI)	p-value*
	Before	After		
OHIP-14	9.4±6.4	5.2±6.0	4.2 (2.7 to 5.8)	<0.001

*Paired t-test

## Discussion

Dental bleaching using a gingival barrier is routine in dental offices. In a clinical scenario with in-office bleaching using high concentrations of HP, there is no doubt about the effectiveness of using gingival barriers to control GI or even gingival burns in patients. This study was carried out to verify whether GI occurred when a gingival barrier for in-office bleaching with 6% HP was not placed. This is a new scenario, occurring during the popularization of low bleach concentrations in several parts of the world^,2,12,13,30^ mainly in Europe, in which 6% HP is the maximum allowed to be used whether in-office or at-home treatment.^31^

As far as the authors are aware, this is the first study concerned with evaluating GI in relation to the presence or absence of a gingival barrier for low concentrations of bleaching gel. In concentrations up to 10% for at-home bleaching, the gingival barrier may be avoided,^19,32^ and eliminating the step of placing the barrier for in-office bleaching makes the procedure simpler, faster, and less expensive.

GI is of great importance in the evaluation of clinical studies of tooth bleaching. Depending on the degree of GI, it can even manifest ulcerations and burns, being initially described as direct discomfort or pain in the gingival tissue.^9,33^ Unfortunately, in-office bleaching studies rarely assess GI because it is directly associated with the operator’s care for the patient. However, despite several studies having evaluated 6% HP for in-office bleaching,^2,12,17,34-36^only Ferraz, et al.^17^ (2019) evaluated GI. In that study, the author reported GI in 57.7% of participants, differing from this study, which obtained a total of 30.8%. Some methodological differences between Ferraz’s study and this one could help to explain the results in the latter.^17^ The percentage difference can be attributed to the used protocol; the former^17^ used light activation, and light activation of in-office bleaching can increase the operatory temperature,^37,38^thus burning soft tissue.^38^

As previously described, the use of a gingival barrier is effective in controlling GI. However, it is important to note that GI can still occur despite its use. For instance, Al Shethri, et al.^39^ (2003) demonstrated the presence of GI even with the use of the barrier, as did Bruzzell, et al.^9^ (2013) suggesting that this may be attributed to the use of highly concentrated bleaching gels. Notably, one study demonstrated genotoxic potential when bleaching agents in higher concentrations were tested,40 whereas 6% HP neither affected nor altered cell morphology.^41^

GI intensity in both groups was less than 0.3 on a scale of 10, considered clinically insignificant. Unlike the absolute risk of GI, the intensity of GI was similar to that reported in the literature. For instance, in a study by Ferraz, et al.^17^ (2019) low intensity was detected when the gingival barrier was used. Additionally, GI intensity without a gingival barrier was similar to levels determined during at-home bleaching, which uses low concentrations.^19,20^ This leads us to accept the first null hypothesis, facilitating the clinical day-to-day process and possibly reducing costs for the bleaching procedure. It is important to note that our study focused on evaluating GI in the patient’s lower arch. This constitutes part 2 of the research, with part 1 having been previously published.^2^ Part 1 was conducted in the upper arch at a different time.

Various methods were employed for color evaluation in this study, a critical aspect given that the absence of a gingival barrier may lead to contact between the bleaching gel and saliva. It has been previously noted that saliva can interfere with the degradation of HP.^42^ Therefore, evaluating color changes becomes essential in understanding the potential impact of these interactions.^42^ For the objective method, a spectrophotometer was used, which is the least affected by the observer’s training and variability.^43^ For the subjective method, the Vita Classical and Vita Bleachedguide 3D-MASTER scales were used, which facilitate comparisons with previous studies as they were the most used in previous bleaching studies.^12,30,34^ There was no difference between groups in all evaluated parameters, prompting us to accept the second null hypothesis. The results are similar to the study by Carneiro, et al.^2^ (2023) who used the same protocol as the present study in adolescents, even in teeth assessed in different arches. Other studies that evaluated, by the same methods, 6% HP (objective^12,30,34^and subjective methods,^12,30,34,35^) demonstrated similar bleaching efficacy. However, it is worth mentioning that the protocol used in this study, as well as that by Carneiro, et al.^2^ (2023) and Bersezio, et al.^34^ (2019) used no light and obtained similar results to the studies that used it.^12,30^ The bleaching pattern was acceptable when compared to studies that used higher concentrations of HP.^36,44^ However, one more session could be undertaken to achieve better results since the use of HP products in low concentrations can produce the same efficacy of color change with the advantage of having fewer adverse effects.^11^

Assessing patients’ quality of life in clinical studies is of great importance. Due to the fact that it is a result reported by the patient, its evaluation is already considered essential because it demonstrates how much oral health influences patients’ lives.^45^Adolescents are concerned about social acceptance and appearance, and negative psychosocial judgments have been demonstrated in children and adolescents from the age of 11.^46^ Thus, aesthetic procedures, such as tooth bleaching, can increase a patient’s self-esteem and emotional well-being,1 as well as quality of life. This study demonstrated a significant improvement in the effects of the oral condition on patients’ quality of life after bleaching, thus prompting us to reject the third null hypothesis. The results presented are in line with other studies that used 6% HP.^12,34,35^

It is crucial to emphasize that there is a significant demand for orthodontic treatments, including tooth bleaching, in the adolescent age group.^47^ Therefore, following the bleaching procedure in these patients, it is essential to consider waiting for bracket bonding to avoid potential inconveniences. Changes occurring in the dental substrate could lead to bracket detachment.^47,48,49^ However, it is important to note that there is a lack of clinical studies in the literature to confirm these hypotheses.

Some limitations of the study need to be described. The sample size for detecting a difference smaller than the proposed one was relatively small. Additionally, this study included no visual assessment by the clinician evaluating GI. It tested only one brand of bleaching gel available on the market. Moreover, this research employed only one protocol for the use of HP 6% in in-office bleaching, strictly adhering to the manufacturer’s recommendations. Results are unable to be directly applied for the over-the-counter or at-home bleaching materials based on 6% HP, mainly because protocols related to the use of 6% HP vary widely.^12,17,30,34-36^ Therefore, future clinical studies with larger designs need to be done to evaluate if GI is affected at the same level if different brands and/or application protocols of bleaching materials are used.

## Conclusions

The use or not of the gingival barrier for in-office bleaching with 6% HP in adolescents proved to be equivalent for gingival irritation. A significant color change was observed in both groups. Bleaching with 6% HP improved the impact of oral condition on patients’ quality of life. Therefore, the step of applying the gingival barrier for 6% HP in in-office bleaching can be disregarded.
